# A Novel Mathematical Approach to Gait Analysis: The Reliability and Validity of the ZAY Angle for Step Length Estimation in Healthy Adults

**DOI:** 10.3390/s25072142

**Published:** 2025-03-28

**Authors:** Ziad M. Haroun, Hoda M. Zakaria, Zizi M. Ibrahim, Osama R. Abdelraouf, Aya A. Khalil

**Affiliations:** 1 Department of Musculoskeletal Disorders, Faculty of Physical Therapy, Innovation University, 10th of Ramadan City 7055501, Egypt; ziad.haroun9899@gmail.com; 2 Department of Neuromuscular Disorders and Its Surgery, Faculty of Physical Therapy, Cairo University, Cairo 12612, Egypt; dr.hodazakaria@yahoo.com; 3 Department of Rehabilitation Sciences, College of Health and Rehabilitation Sciences, Princess Nourah bint Abdulrahman University, P.O. Box 84428, Riyadh 11671, Saudi Arabia; zmibrahim@pnu.edu.sa; 4 Physical Therapy Program, Batterjee Medical College, Jeddah 21442, Saudi Arabia; pt4.jed@bmc.edu.sa; 5 Department of Biomechanics, Faculty of Physical Therapy, Cairo University, Cairo 12612, Egypt; 6 Department of Biomechanics, Faculty of Physical Therapy, Horus University-Egypt, New Damietta 34517, Egypt

**Keywords:** gait assessment, mathematical formula, model, step length

## Abstract

**Highlights:**

**What are the main findings?**

- The utilization of smartphone applications to assess gait kinematics yields reliable data.
- The ZAY angle is a valid and reliable angle that can be used to estimate individualized step lengths.

**What is the implication of the main findings?**

- Application of the arc length formula might provide an alternative method to determine individualized step lengths.
- Clinicians could include the ZAY angle in their gait analysis profiles to obtain individualized step lengths.

**Abstract:**

(1) Background: The application of a mathematical formula to human gait at certain phases is a considerable method to avoid the issues associated with complicated procedures of gait assessment. The purpose of this study was to identify the validity and reliability of an angle (the ZAY angle) in estimating and predicting the step length in healthy subjects. (2) Methods: Thirty-three college-aged students participated in this study. For an assessment of each participant’s gait, a 4.5 m walkway was covered with a weight paper roll to mark the participant’s footprints, providing the step lengths of six consecutive steps for two trials. At the same time, a video recording was captured and analyzed by the Coach’s Eye application to determine the step angle (ß). The arc length formula was utilized to calculate the ZAY angle (θ). Spearman’s rho correlation coefficient and the interclass correlation coefficient were used to test the validity and reliability of the ZAY angle in determining individualized step lengths in healthy subjects. Simple linear regression was used to test if the calculated angle could significantly predict the step length. (3) Results: The Spearman rho correlation between the analyzed and calculated angles was significant for all three step lengths (*p* < 0.05). It was found that the calculated angle could significantly predict the step length (β = 0.91, *p* < 0.05). The ICC was very high (*p* < 0.05). (4) Conclusions: The ZAY angle is a valid and reliable angle that can be used to estimate individualized step lengths. Clinicians could include this angle in their gait analysis profiles to achieve individualized assessment and rehabilitation goals.

## 1. Introduction

Spatial gait parameters are crucial clinical indicators that reflect the functional status and can help forecast potential negative health outcomes [1]. Evaluating gait in typical conditions offers important insights into an individual’s physical well-being. One of the most commonly utilized gait descriptors for quantitative and objective evaluations is the step length (SL). The SL is defined as the distance between the initial contact point of one foot and the initial contact point of the contralateral foot [2]. Step length asymmetry is a significant clinical indicator for unilateral lower limb pathologies such as amputation, hemiparesis, and osteoarthritis. A reduced step length is a broad indicator of an underlying gait disturbance, particularly in neurological conditions such as Parkinson’s disorders [3] and other rare neurological diseases [4].

Recent advancements in computational gait analysis have further demonstrated the diagnostic potential of machine learning techniques. For example, the work of Ji et al. [5] showed how integrating diverse gait parameters with artificial intelligence can enhance the identification of specific neurological impairments, providing a pathway for early and precise clinical interventions. Meanwhile, theoretical frameworks challenge traditional models by proposing that embodied dynamics are central to gait rhythm formation, emphasizing the interplay between biomechanical structures and environmental interactions rather than rigid algorithmic encoding [6]. These innovations emphasize the growing integration of computational techniques with theoretical frameworks in gait analysis.

The step length can be determined using model-based approaches or empirical methods [7]. Empirical methods depend on the relationships between the SL and various factors, such as the acceleration magnitude [8], the acceleration variance and step frequency [9], and the disparity between the minimum and maximum vertical acceleration [10]. Zijlstra and Hof’s [11] inverted pendulum model of human gait, a model-based approach, emphasizes the connection between the SL and the vertical displacement of the body’s center of mass. Gonzalez et al. [12] refined this model by incorporating the double-stance phase as a function of foot length.

Temporospatial parameters (TSPs), which are influenced by anthropometric factors like height and limb length, are often standardized using leg length to account for individual variability [13]. Hoff’s early work [14] highlighted the necessity of normalizing step length measurements to limb length, as differences in height and lower extremity proportions complicate cross-individual comparisons.

Gait analysis in the sagittal plane is relatively straightforward, but multi-planar or force-related assessments require specialized 3D systems. Biomechanics researchers seek to establish consistent parameters for human movement that are comprehensible, comparable, and easily shared within the scientific community [15]. While laboratory-based 3D motion analysis remains the gold standard, its cost and accessibility limitations have spurred the development of affordable alternatives [16].

Integrating technology into healthcare is essential. Smartphones offer a promising means for managing health services thanks to their ease of use, high acceptability, and appropriate technical features [7]. The use of smartphone applications (SPAs) enables low-cost and easy quantitative assessments of gait [16]. Applications like Coach’s Eye simplify joint angle analysis, offering accessible solutions for remote or resource-limited settings [17].

**Figure 1:**
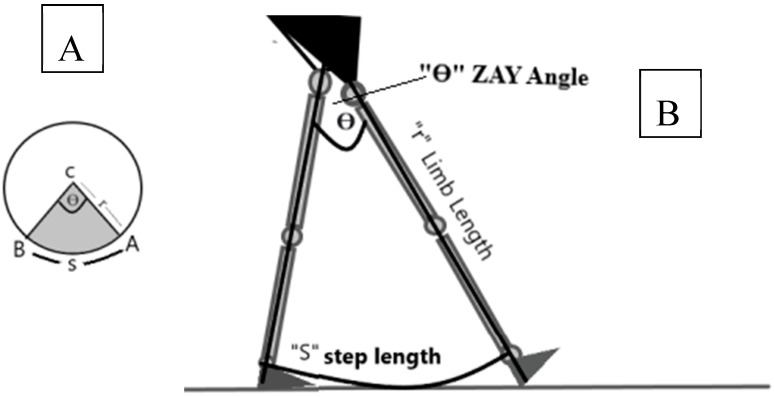
The application of the arc length formula to human gait. (**A**) The arc length formula. (**B**) The application of the formula to human lower extremities at initial contact.

In this study, we propose a geometric model for step length calculation during the initial contact phase of gait. By conceptualizing the hip joint as the center of a circle with the limb length as the radius, the step length corresponds to the arc length determined by the angle between the successive limbs (Figure 1). This model integrates the limb length and angular displacement into a single equation (θ = X·180/π·R), aiming to individualize step length measurements while circumventing the complexities of traditional gait analysis. Although the long-term goal is to apply this method to pathological gaits, validation in healthy populations is a critical first step. This study evaluated the reliability and validity of the proposed formula using the Coach’s Eye SPA; examined the relationships between step length, limb length, and angular variables; and tested whether the calculated angle significantly predicted the step length.

The formula for calculating the arc length in radians is given by arc length = θ × r, where θ is in radians. Arc length = θ × (π/180) × r, where θ is in degrees and the variables are as follows:

- S = the length of the arc = the step length.
- θ = the central angle of the arc = the ZAY angle = the angle between one hip at initial contact and the other hip in the terminal stance before the toes lift off the ground.
- r = the radius of the circle = the limb length.

## 2. Materials and Methods

### 2.1. Design

This study was designed to investigate the validity and reliability of the ZAY angle. To determine the test–retest reliability, each participant walked twice with a five-minute interval. To assess the validity, the data derived from the ZAY angle were compared to those measured by the Coach’s Eye SPA.

### 2.2. Participants

Thirty-three college-aged students (16 males and 17 females) at Cairo University in Egypt participated in this study. Their age, body mass, and height values (means ± SDs) were 22.48 ± 2.40 years, 67.79 ± 10.85 kg, and 170.36 ± 10.35 cm, respectively. The participants had full ROM of the ankle, knee, and hip and normal foot posture. Individuals were excluded from this study if they had undergone any lower extremity surgeries or experienced fractures within the six months leading up to their enrollment [18]. Other exclusion criteria included leg length discrepancies and scoliosis.

### 2.3. Ethical Approval

Every participant signed a written consent form prior to this study. Ethical approval was provided by the Ethics Committee of the Faculty of Physical Therapy, Cairo University (No.: P.T.REC/012/004412). This study followed the principles outlined in the Declaration of Helsinki.

### 2.4. Sample Size Calculation

A power analysis was conducted in G*Power 3.1 software to estimate the necessary sample size for detecting a moderate correlation (effect size: r = 0.55) between the angle measurements derived from the Coach’s Eye application and those calculated using the proposed equation during the initial three steps. Employing a bivariate normal correlation model with a significance level (α) of 0.05 and 80% statistical power, the analysis yielded a minimum required sample size of 19 participants. The calculated sample size of 19 participants aligned with established empirical guidelines for correlational studies, which recommend a minimum of 15–20 subjects to reliably detect moderate effects (r > 0.5) [19,20].

### 2.5. Instrumentation

#### 2.5.1. Camera System

Two mobile devices, specifically(Samsung A03S devices, Suwon, Republic of Korea) equipped with a triple camera system consisting of a 13-megapixel (f/2.2) primary lens, a 2-megapixel (f/2.4) camera, and another 2-megapixel (f/2.4) camera, were utilized to record the participants’ body movements as they walked along the path. A video capturing the walking gait in the sagittal plane was recorded 2.7 m from the right side of the walkway at a height of 90 cm, ensuring the subject’s entire body was visible throughout the recording. The cameras were spaced 2.5 m apart [17].

#### 2.5.2. Coach’s Eye (TechSmith Corporation, East Lansing, MI, USA, Version 5)

Coach’s Eye is a mobile app designed for 2D motion analysis, and it can evaluate gait in healthy individuals and patients. However, its primary purpose is to help trainers and coaches evaluate athletic performance. This application calculates joint angles and their changes using a digital goniometer, eliminating the need to place any markers on the body. Videos can be captured from the frontal and sagittal perspectives and analyzed frame by frame, either backward or forward. A digital video library enables the comparison of recorded footage with that of other athletes [21]. Previous research indicates that Coach’s Eye is capable of delivering accurate and dependable kinematic assessments of both gait and running [7,17].

### 2.6. Procedures

**Figure 2:**
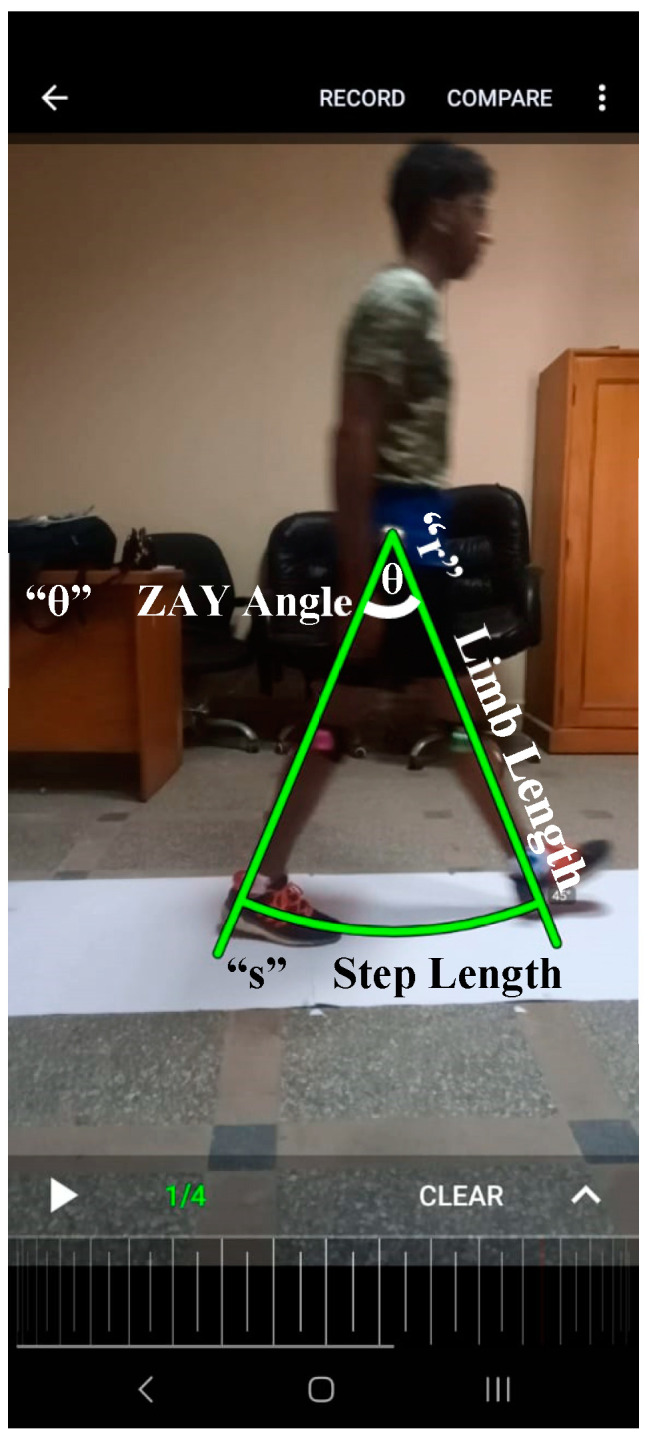
A screenshot of a participant’s gait analyzed by the Coach’s Eye application with superimposed lines indicating how to apply the formula to obtain the ZAY angle.

This study aimed to assess the reliability and validity of the equation θ = (S·180/π·R) when calculating step length using the Coach’s Eye SPA. For the testing procedures, a 4.5 m walkway covered with a weight paper roll was used in the Biomechanics laboratory of the Faculty of Physical Therapy, Cairo University. Each participant’s lower limb length (m), described as the space from the greater trochanter to the end of the shoes, was measured from the side while the participants were standing. The end of the shoes was used as the last point of contact with the ground on the same line as the markers on the limb. The soles of the participants’ shoes were colored with dark watercolors to make footprints. The participants were recorded with a video camera for six consecutive steps two times with a five-minute interval. Then, each step length was measured, and θ, “the angle between both hips”, was calculated using the equation. Videos analyzed using Coach’s Eye were used to determine the same angle (ß) at the moment when one hip was at initial contact and the other was in the terminal stance (Figure 2). The participants were asked to wear comfortable clothes and shoes.

Two mobile cameras were used to record the gaits of the participants.

Five markers were positioned on distinct anatomical reference points, including the right greater trochanter, right lateral malleolus, left medial malleolus, lateral knee joint line, and left medial knee joint line. The 4.5 m walkway using paper rolls on the floor was fixed with glue. The soles of the participants’ shoes were colored with watercolors before walking on the walkway to make footprints, allowing the length of each step to be measured as we recorded a video with a mobile camera to cover the full distance of the gait [15].

The step lengths of the footprints were measured by a tape measure (from the initial contact of one footprint to the initial contact of the next footprint along the paper roll), and the ZAY angle “θ” (the step length angle) was calculated.

The ZAY angle = the angle between one hip at initial contact and the other hip in the terminal stance before the toes lift off the ground.

θ=(S·180)/(π·R)

where

“θ” = the ZAY angle.

S = step length.

R = limb length.

This equation was taken from the arc length formula.

The length of an arc can be determined using various formulas, depending on the measurement unit of the arc’s central angle. The central angle can be expressed in either radians or degrees, and consequently, the length of the arc of a circle is determined. For a circle, the formula to determine the arc length is θ times the circle’s radius. Figure 1 shows the application of the equation to a human gait where the hip joint is the center of the circle, the limb length is the radius of the circle, the angle between one hip at initial contact and the other hip in the terminal stance is the central angle of the arc, and the step length is the length of the arc.

In this example, the participant’s limb length was 92.5 cm and the step length was 74 cm. By applying the formula, in this case the angle = (74 × 180)/(π × 92.5) = 45.8 degrees. When measuring the same angle by the application, the angle = 45 degrees.

### 2.7. Data Analysis

The Spearman rho correlation was calculated for the angle assessed by the Coach’s Eye application and the angle calculated with the introduced equation for the first three steps. In addition, the relationships between the step length and the limb length, calculated angle, and analyzed angle were studied. A Bland–Altman graph with 95% limits of agreement was plotted to compare the calculated and analyzed angles. To analyze the correlation between the step length and the computed angle, a single linear regression analysis was constructed. The calculated angle was considered the predictor, and the step length was the dependent variable.

The participants walked six steps two times; the first steps in the two trials were used to evaluate test–retest reliability. The Intraclass Correlation Coefficient (ICC) was computed to assess test–retest reliability, along with the 95% confidence intervals (CIs) between the initial steps of the two trials. The reliability was deemed to be excellent (0.91–1.00), substantial (0.76–0.90), moderate (0.41–0.75), or weak (0.00–0.40) [22].

All evaluations were performed using the Statistical Package for Social Sciences™ (Version 24, IBM Corp., Armonk, NY, USA). All analyses were conducted with the alpha level set at 0.05.

## 3. Results

### 3.1. Validity

**Figure 3:**
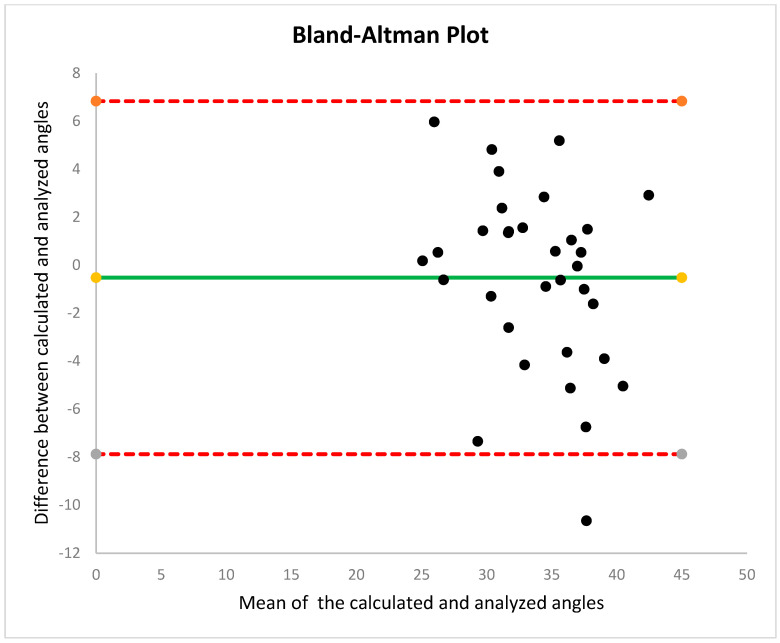
A Bland–Altman plot with 95% limits of agreement to compare the calculated and analyzed angles.

The descriptive statistics of the participants’ kinematic gait parameters are presented in Table 1. The Spearman rho correlation between the analyzed and calculated angles was significant for all three step lengths (*p* < 0.001). The Spearman rho correlation coefficient was strong (Table 2). The Bland–Altman plot revealed comparable patterns between the angles, with values near the average, consistent homogeneity, and decreased dispersion within the agreement limits (Figure 3).

**Figure 4:**
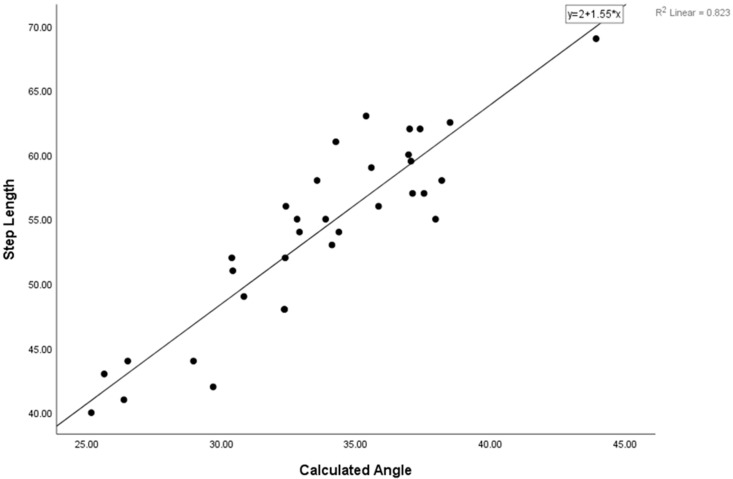
The fitted regression model found that the calculated angle could significantly predict the step length.

The relationships between the step length and the limb length (moderate), calculated angle (very high), and analyzed angle (high) were significant (*p* < 0.05) (Table 3). Simple linear regression was used to test if the calculated angle significantly predicted the step length. The fitted regression model was step length = 1.5 + (0.91 × (calculated angle)). The overall regression was statistically significant (R2 = 0.823, F (1, 31) = 144.36, *p* = 0.0001). It was found that the calculated angle could significantly predict the step length (β = 0.91, *p* = 0.0001) (Figure 4).

### 3.2. Reliability

The ICC was very high (*p* < 0.05) (Table 4).

## 4. Discussion

Human gait patterns serve as critical biomarkers of individual biomechanical function and quality of life. The ability to reliably quantify gait characteristics, including spatiotemporal parameters and kinematics, has become indispensable across clinical, rehabilitative, athletic, and robotic applications [23]. While traditional gait analysis often requires sophisticated laboratory equipment, recent advancements in smartphone applications (SPAs) have enabled portable, cost-effective kinematic assessments in both field and laboratory settings [23]. This study introduces a novel geometrical model based on the arc length formula to estimate individualized step lengths, addressing the logistical and technical challenges of gait analysis in resource-limited environments. Below, we contextualize these findings within the broader literature, highlighting methodological innovations and unresolved gaps.

The proposed model utilizes the arc length formula to calculate the step length, employing the limb length and the step angle (the ZAY angle), which demonstrates high test–retest reliability when validated against the Coach’s Eye SPA. This approach aligns with recommendations by the authors of [24], who emphasized the need to normalize gait parameters to anthropometric factors (e.g., limb length) to mitigate bias from inter-individual variability [25]. Unlike prior studies that derived step length from theoretical parameters [26] or walking speed and step frequency [3,27], our model integrates limb length and the hip joint angle into a single equation, a methodological departure that enhances personalization. This innovation addresses a critical gap identified by Allseits et al. [1], whose inverted pendulum model utilized inertial measurement units (IMUs) to estimate step length based on thigh and shank angular velocities but did not account for hip kinematics. Allseits et al. [1] focused on circumventing challenges in double-limb calculations.

Our findings corroborate existing evidence that the step length correlates strongly with the hip flexion angle [28,29]. Schulz et al. [30] similarly identified hip and torso kinematics as key determinants of step length, though their analysis emphasized kinetic forces rather than angular relationships. Notably, Whitcome et al. [31] reported gender-specific adaptations in gait strategies: women achieve comparable walking speeds to men through greater hip flexion angles rather than increased stride frequency, compensating for shorter limb lengths. This aligns with our model’s emphasis on individualized adjustments, suggesting that anatomical variations necessitate tailored kinematic solutions. Furthermore, our observations of energy-efficient asymmetric walking patterns in healthy adults [32] underscore the adaptive nature of human locomotion, reinforcing the need for personalized gait metrics.

A central challenge in gait rehabilitation lies in defining “normal” movement patterns. Hollman et al. [33] highlighted the variability in normative spatiotemporal parameters across populations, a limitation compounded by the scarcity of data on gait variability metrics [34]. Our model’s ability to estimate step length via the ZAY angle offers a practical tool for creating individualized participant profiles, potentially supplementing population-based references. This is particularly relevant given the lack of consensus on pathological versus normal gait thresholds [34], a gap our methodology seeks to narrow.

The current study has some limitations. First, the results are limited to healthy college-aged participants; caution should be taken when replicating this study with older individuals, and studies involving patient groups will undoubtedly be necessary. To evaluate test–retest reliability, the participants completed two walking sessions with a five-minute rest in between, whereas many studies typically allow a few days between the original test and the retest. Additionally, the markers were not taken off between the first and second tests. These limitations could influence how our findings compare to other research. Fourth, we employed the Coach’s Eye app and ground footprints to measure the step length and hip angle. However, Sessoms [35] studied walking and manipulated the step length using floor markers with a similar methodology.

Regarding the accuracy of the Coach’s Eye SPA, Mousavi et al. [17] examined the accuracy and consistency of the Coach’s Eye SPA in analyzing lower-limb kinematics while running on a treadmill, comparing its findings with those obtained from a conventional 3D motion analysis system (Vicon). Regarding its validity, Coach’s Eye displayed a variance of only 1–2 degrees in kinematic measurements for sagittal-plane hip angles when compared to Vicon. Additionally, Coach’s Eye exhibited strong consistency in test–retest reliability for joint kinematic measurements, aligning with the findings of Krause et al. [36], who noted the application’s high reliability during squat execution [15].

## 5. Conclusions

The current study revealed that the arc length formula could estimate step length with excellent test–retest reliability. The ICC for the validity of the ZAY angle and the analyzed angle from the Coach’s Eye app was strong. The ZAY angle can therefore be used as an alternative method for estimating step length during walking.

The need for an individualized step length under normal conditions is crucial for deriving comparisons during pathology. Gait assessment laboratories are expensive and may be inaccessible. The application of the formula might provide an alternative method for determining individualized step lengths.

We encourage individuals to perform gait assessments using the equation to obtain their own reference data. Our methodology is simple, valid, and reliable, making it suitable for reproduction in future studies on abnormal conditions.

## Figures and Tables

**Table 1:** Participants’ kinematic gait parameters.

Gait Parameter	Mean ± SD
Limb length	92.08 ± 5.18 cm
Step Length (X1)	53.94 ± 7.19 cm
Step Length (X2)	59.68 ± 8.67 cm
Step Length (X3)	61.73 ± 7.71 cm
Calculated Angle (θ1)	33.57 ± 4.21
Analyzed Angle (ß1)	34.09 ± 5.25
Calculated Angle (θ1)	35.16 ± 4.67
Calculated Angle (θ2)	37.13 ± 4.99
Analyzed Angle (ß2)	39.76 ± 4.83
Calculated Angle (θ3)	38.41 ± 4.45
Analyzed Angle (ß3)	40.03 ± 5.69

**Table 2:** The correlations between the analyzed angle and the calculated angle for step lengths 1, 2, and 3.

Correlations	r	*p* Value
Analyzed angle vs. calculated angle 1	0.70	0.001 *
Analyzed angle vs. calculated angle 2	0.66	0.001 *
Analyzed angle vs. calculated angle 3	0.60	0.001 *

* indicates significance (*p* < 0.5).

**Table 3:** The correlations among the step length and the limb length, calculated angle, and analyzed angle.

Variables	Step Length r	*p* Value
Limb Length	0.35	0.04 *
Calculated Angle (θ1)	0.87	0.001 *
Analyzed Angle (ß1)	0.60	0.001 *

* indicates significance (*p* < 0.5).

**Table 4:** Reliability statistics.

**Test–retest reliability between two trials**	**ICC**	*p* value = 0.001 *
Calculated angle of first step in trial 1 vs. trial 2	0.81	

* indicates significance (*p* < 0.5).

## Data Availability

Data are available upon request.

## Abbreviations

The following acronyms are used in this manuscript:

SL	step length
SPAs	smartphone applications
ICC	Intraclass Correlation Coefficient
CIs	confidence intervals
TSPs	temporospatial parameters
IMUs	inertial measurement units

## References

[1] Allseits E., Agrawal V., Lučarević J., Gailey R., Gaunaurd I., Bennett C. (2018). A practical step length algorithm using lower limb angular velocities. J. Biomech..

[2] Park J.W., Baek S.H., Sung J.H., Kim B.J. (2022). Predictors of Step Length from Surface Electromyography and Body Impedance Analysis Parameters. Sensors.

[3] Zijlstra A., de Bruin E.D., Bruins N., Zijlstra W. (2008). The step length–frequency relationship in physically active community-dwelling older women. Eur. J. Appl. Physiol..

[4] Indelicato E., Raccagni C., Runer S., Hannink J., Nachbauer W., Eigentler A., Amprosi M., Wenning G., Boesch S. (2022). Instrumented gait analysis defines the walking signature of CACNA1A disorders. J. Neurol..

[5] Ji X., Zeng W., Dai Q., Zhang Y., Du S., Ji B. (2023). Machine learning-based detection of cervical spondylotic myelopathy using multiple gait parameters. BIROB.

[6] Li L., Ma S., Tokuda I., Liu Z., Ma Z., Tian Y., Kang S. (2024). Embodying rather than encoding: Towards developing a source-filter theory for undulation gait generation. BIROB.

[7] Pepa L., Verdini F., Spalazzi L. (2017). Gait parameter and event estimation using smartphones. Gait Posture.

[8] Kim J.W., Jang H.J., Hwang D.H., Park C. (2004). A step, stride and heading determination for the pedestrian navigation system. J. Glob. Position. Syst..

[9] Shin S.H., Park C.G. (2011). Adaptive step length estimation algorithm using optimal parameters and movement status awareness. Med. Eng. Phys..

[10] Weinberg H. (2002). Using the ADXL202 in pedometer and personal navigation applications. Analog Devices AN-602 Appl. Note.

[11] Zijlstra W., Hof A.L. (1997). Displacement of the pelvis during human walking: Experimental data and model predictions. Gait Posture.

[12] Gonzalez R.C., Alvarez D., Lopez A.M., Alvarez J.C. (2007). Modified pendulum model for mean step length estimation. Proceedings of the 2007 29th Annual International Conference of the IEEE Engineering in Medicine and Biology Society.

[13] Kirtley C. (2006). Clinical Gait Analysis: Theory and Practice.

[14] Hof A.L. (1996). Scaling gait data to body size. Gait Posture.

[15] Roggio F., Ravalli S., Maugeri G., Bianco A., Palma A., Di Rosa M., Musumeci G. (2021). Technological advancements in the analysis of human motion and posture management through digital devices. World J. Orthop..

[16] Manor B., Yu W., Zhu H., Harrison R., Lo O.Y., Lipsitz L., Zhou J. (2018). Smartphone app–based assessment of gait during normal and dual-task walking: Demonstration of validity and reliability. JMIR mHealth uHealth.

[17] Mousavi S.H., Hijmans J.M., Moeini F., Rajabi R., Ferber R., van der Worp H., Zwerver J. (2020). Validity and reliability of a smartphone motion analysis app for lower limb kinematics during treadmill running. Phys. Ther. Sport.

[18] Tanen L., Docherty C.L., Van Der Pol B., Simon J., Schrader J. (2014). Prevalence of chronic ankle instability in high school and division I athletes. Foot Ankle Spec..

[19] Bujang M.A., Baharum N. (2017). A simplified guide to determination of sample size requirements for estimating the value of intraclass correlation coefficient: A review. Arch Orofac. Sci..

[20] Faul F., Erdfelder E., Lang A.G., Buchner A. (2007). G Power 3: A flexible statistical power analysis program for the social, behavioral, and biomedical sciences. Behav. Res. Methods.

[21] Whiteley R. (2015). Coach’s eye. Br. J. Sports Med..

[22] Ko J., Rosen A.B., Brown C.N. (2018). Functional performance deficits in adolescent athletes with a history of lateral ankle sprain(s). Phys. Ther. Sport.

[23] Akhtaruzzaman M., Shafie A.A., Khan M.R. (2016). Gait analysis: Systems, technologies, and importance. J. Mech. Med. Biol..

[24] Valencia O., Araneda O., Cárcamo M., Carpes F.P., Venegas R.G. (2018). Relationship between lower limb anthropometry and temporo-spatial parameters in gait of young adults. Retos.

[25] Andrews J.R., Harrelson G.L., Wilk K.E. (2012). Physical Rehabilitation of the Injured Athlete: Expert Consult-Online and Print.

[26] Sun Y., Wu H., Schiller J. (2015). A step length estimation model for position tracking. Proceedings of the 2015 International Conference on Localization and GNSS (ICL-GNSS).

[27] Yang S., Li Q. (2010). Ambulatory walking speed estimation under different step lengths and frequencies. Proceedings of the 2010 IEEE/ASME International Conference on Advanced Intelligent Mechatronics.

[28] Sawicki G.S., Ferris D.P. (2009). Powered ankle exoskeletons reveal the metabolic cost of plantar flexor mechanical work during walking with longer steps at constant step frequency. J. Exp. Biol..

[29] Lim Y.P., Lin Y.C., Pandy M.G. (2017). Effects of step length and step frequency on lower-limb muscle function in human gait. J. Biomech..

[30] Schulz B.W., Ashton-Miller J.A., Alexander N.B. (2008). The effects of age and step length on joint kinematics and kinetics of large out-and-back steps. Clin. Biomech..

[31] Whitcome K.K., Miller E.E., Burns J.L. (2017). Pelvic rotation effect on human stride length: Releasing the constraint of obstetric selection. Anat. Rec..

[32] Roemmich R.T., Leech K.A., Gonzalez A.J., Bastian A.J. (2019). Trading symmetry for energy cost during walking in healthy adults and persons poststroke. Neurorehabilit. Neural Repair.

[33] Hollman J.H., McDade E.M., Petersen R.C. (2011). Normative spatiotemporal gait parameters in older adults. Gait Posture.

[34] Beauchet O., Allali G., Sekhon H., Beauchet O., Allali G., Sekhon H., Verghese J., Guilain S., Steinmetz J.P., Helbostad J.L. (2017). Guidelines for assessment of gait and reference values for spatiotemporal gait parameters in older adults: The biomathics and Canadian gait consortiums initiative. Front. Hum. Neurosci..

[35] Sessoms P.H. (2008). Step by step: A study of step length in able-bodied persons, race walkers, and persons with amputation. Ph.D. Thesis.

[36] Krause D.A., Boyd M.S., Hager A.N., Smoyer E.C., Thompson A.T., Hollman J.H. (2015). Reliability and accuracy of a goniometer mobile device application for video measurement of the functional movement screen deep squat test. Int. J. Sports Phys. Ther..

